# Distribution, Biosynthesis, and Function of Carotenoids in Oxygenic Phototrophic Algae

**DOI:** 10.3390/md23020062

**Published:** 2025-01-31

**Authors:** Shinichi Takaichi

**Affiliations:** Department of Molecular Microbiology, Faculty of Life Sciences, Tokyo University of Agriculture, Sakuragaoka, Setagaya, Tokyo 156-8502, Japan; shintakaichi@gmail.com

**Keywords:** algal phylogeny, biosynthesis of carotenoids, distribution of carotenoids, distribution of chlorophylls, function of carotenoids, pigment-protein complexes

## Abstract

For photosynthesis, oxygenic phototrophic organisms necessarily contain not only chlorophylls but also carotenoids. Various carotenoids have been identified in algae and taxonomic studies of algae have been conducted. In this review, the relationship between the distribution of chlorophylls and carotenoids and the phylogeny of sea and freshwater oxygenic phototrophs, including cyanobacteria, red algae, brown algae, and green algae, is summarized. These phototrophs contain division- or class-specific chlorophylls and carotenoids, such as fucoxanthin, peridinin, diadinoxanthin, and siphonaxanthin. The distribution of β-carotene and its derivatives, including β-carotene, zeaxanthin, violaxanthin, neoxanthin, diadinoxanthin, fucoxanthin, and peridinin (β-branch carotenoids), are limited to divisions of a part of Rhodophyta, Cryptophyta, Heterokontophyta, Haptophyta, and Dinophyta. Meanwhile, the distribution of α-carotene and its derivatives, such as lutein, loroxanthin, and siphonaxanthin (α-branch carotenoids), are limited to divisions of a part of Rhodophyta (macrophytic type), Cryptophyta, Euglenophyta, Chlorarachniophyta, and Chlorophyta. In addition, carotenogenesis pathways are also discussed based on the chemical structures of carotenoids and the known characteristics of carotenogenesis enzymes in other organisms. The specific genes and enzymes for carotenogenesis in algae are not yet known. Most carotenoids bind to membrane-bound pigment-protein complexes, such as reaction centers and light-harvesting complexes. Some carotenoids function in photosynthesis and are briefly summarized. Water-soluble peridinin-chlorophyll *a*-protein (PCP) and orange carotenoid protein (OCP) have also been characterized. This review is a summary and update from the previous review on the distribution of major carotenoids, primary carotenogenesis pathways, and the characteristics of carotenogenesis enzymes and genes.

## 1. Introduction

Oxygenic phototrophs are classified throughout many divisions of the plant kingdom and bacteria, including cyanobacteria, red algae, brown algae, green algae, and land plants (Table 1). The sizes of algae range from single cells of picophytoplankton—the smallest of which is less than 1 µm—to macrophytic seaweeds—the largest of which is greater than 50 m. Attempts have been made to cultivate single-cell algae and cyanobacteria for a long time, but the number of species that can be cultured was initially limited. With the recent development of culture techniques, some single cells may be cultured, enabling the study of their characteristics, including pigments and phylogeny. The development of taxonomic technologies, such as the DNA base sequences of 16S or 18S rRNA genes, some other genes, additional nuclear and chloroplast genomic DNA, algae phylogenetics, and precise morphological and ultrastructural characteristics, have significantly advanced algal phylogenetics, including eukaryotic algae and cyanobacteria [1,2].

More than 850 structurally defined natural carotenoids have been identified in bacteria, including cyanobacteria and anoxygenic phototrophic bacteria, archaea, fungi, algae, land plants, and animals [2,6]. Except for animals, these organisms can synthesize a variety of carotenoids from commonly isopentenyl pyrophosphate (IPP), and carotenoids are synthesized through diverse carotenogenesis pathways. Their distribution is division- and/or class-specific (Table 1) [1,2,7,8,9,10]. In addition, the characteristics of carotenogenesis genes and enzymes have been examined. Some carotenogenesis genes exhibit high amino acid sequence similarity from bacteria to land plants, whereas some have low similarity. Some homologous genes have been proposed, but some carotenogenesis genes and enzymes, particularly in algae, have not been found.

**Figure 1:**
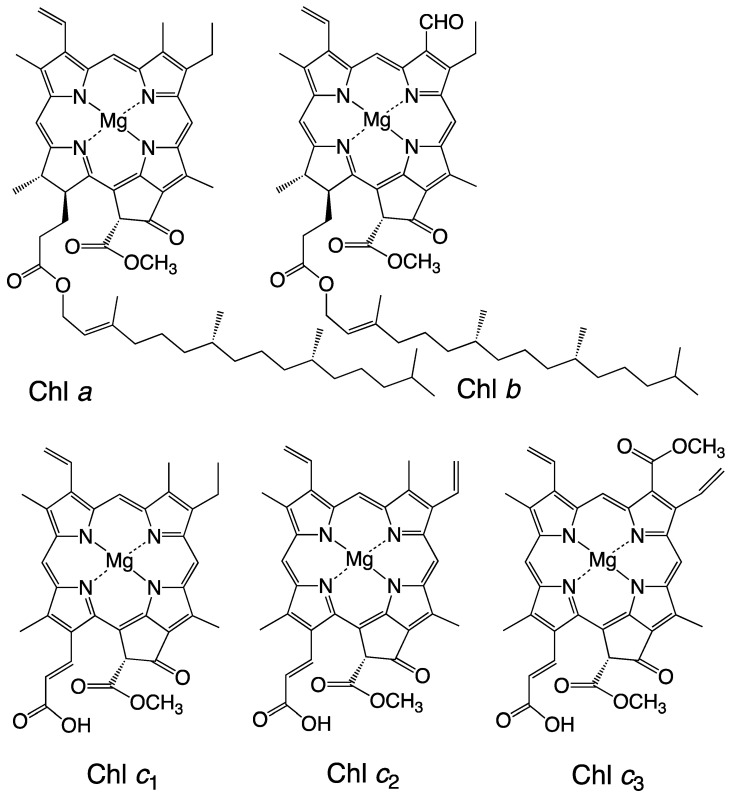
Structures of chlorophylls (Chls).

In contrast to carotenoids, the numbers of chlorophyll (Chl) molecules are limited. Their distribution is division- and/or class-specific, and their biosynthesis has been established (Table 1). The biosynthetic pathways of chlorophylls are common, and each chlorophyll and bacteriochlorophyll is branched from the main pathway [8,9].

Takaichi [3] provided a summary of “Carotenoids in Algae: Distribution, Biosyntheses and Functions” more than one decade ago. Now, this review is updated from the previous one [3]. Oxygenic phototrophs, including algae and cyanobacteria, as well as land plants for comparison, are discussed. This review summarizes the distribution of carotenoids and chlorophylls, carotenogenesis enzymes and pathways, and the function of carotenoids in photosynthesis in oxygenic phototrophs.

## 2. Distribution of Carotenoids and Chlorophylls

Many different carotenoids are found in oxygenic phototrophs, including cyanobacteria, red algae, brown algae, green algae, and land plants. The chemical structures of several important carotenoids in the oxygenic phototrophs are illustrated in the figures of this review. Of these, approximately 30 to 50 types are believed to have direct roles in photosynthesis, whereas others may be intermediates in carotenogenesis or as accumulated carotenoids. Some carotenoids are unique to specific algal divisions and/or classes, making them valuable chemotaxonomic markers (Table 1) [1,2,7,8,9,10].

Carotenoids can be divided into carotenes and xanthophylls. Carotenes consist of only carbon and hydrogen. Xanthophylls contain additionally one or more oxygen, which are usually hydroxy, keto, and/or epoxy groups. They are usually bound to pigment-protein complexes and related to functions as described in Session 4. Hydroxy and keto groups interact with suitable amino acids of peptides to form 3D structures. The Keto group is related to energy transfer. The epoxy group is related to the xanthophyll cycle.

Carotenoids contain several unique functional groups. The allene group (C=C=C) is a unique structure in natural products and is primarily found in carotenoids [11], such as fucoxanthin in brown algae and diatoms, peridinin only in Dinophyta, and 9′-*cis* neoxanthin in green algae, Chlorarachniophyta, and land plants. The acetylene group (C≡C) is a unique structure, and acetylenic carotenoids occur only in algae, such as alloxanthin, crocoxanthin, and monadoxanthin in Cryptophyta, as well as diadinoxanthin and diatoxanthin in Heterokontophyta, Haptophyta, Dinophyta, and Euglenophyta. Acetylated carotenoids (-O-CO-CH_3_), such as fucoxanthin, peridinin, and dinoxanthin, are also primally found in algae, such as Heterokontophyta, Haptophyta, and Dinophyta. These carotenoids are specific to certain algal divisions and classes (Table 1) based on our findings [2,3,4,12,13,14,15] and others [1,6,7,8,9,16,17,18]. The structures of important carotenoids are illustrated in the figures of this review.

On the contrary, the number of chlorophylls is limited. Their structures are relatively similar, and their distribution is also division- and/or class-specific (Table 1). Consequently, the distribution of carotenoids, carotenogenesis pathways, and chlorophylls may be used as valuable chemotaxonomic markers. Their distribution in the oxygenic phototrophs is summarized in Table 1. The structures of chlorophylls are illustrated in Figure 1, and their biosynthetic pathways are summarized in Bryant et al. and Jiang et al. [19,20].

It is believed that oxygenic cyanobacteria occurred from two types of anoxygenic phototrophic bacteria through evolution and endosymbiosis. Whole genome analysis of organisms has provided new insight into the evolution between cyanobacteria and anoxygenic phototrophic bacteria, such as the selective loss model, protocyanobacterium model, and fusion model [21,22,23,24]. Furthermore, all known plastids, including those in algal and land plant chloroplasts, originated from a single primary endosymbiotic event involving a cyanobacterium. This led to eukaryotic cells and the formation of three divisions: Glaucophyta, Rhodophyta, and Chlorophyta. Other algae, such as Cryptophyta, Heterokontophyta, Haptophyta, Euglenophyta, and Chlorarachniophyta, evolved through secondary endosymbiosis of these algae, whereas Dinophyta represents a tertiary endosymbiosis involving some algae [25,26].

Although chlorophylls and their synthetic pathways [19,20] and the peptide components of the photosynthetic pigment–protein complexes in oxygenic phototrophs from cyanobacteria, algae, and land plants, as well as anoxygenic phototrophic bacteria, have remained relatively consistent throughout evolution, the carotenoid compositions have critically changed at each step of endosymbiosis. This suggests that certain pathways and genes for carotenoid synthesis are lost or acquired discontinuously at each evolutionary stage (Table 1) [3,27].

This review describes cyanobacteria found in both seawater and freshwater without distinction since both contain similar pigments. Many cyanobacteria contain β-carotene, zeaxanthin, echinenone, and myxol methylpentosides (myxoxanthophyll), whereas some species lack one or more of these carotenoids. Some contain additional carotenoids, such as nostoxanthin, canthaxanthin, synechoxanthin, and oscillol di-methylpentoside (Table 1) [14]. The carotenoid compositions in cyanobacteria differ from that in chloroplasts of algae. Consequently, during the symbiosis of a cyanobacterium to a eukaryotic cell, carotenoids may undergo considerable restructuring. Notably, since the name “myxoxanthophyll” does not specify the glycoside moieties, we have proposed the name “myxol glycoside” to specify the glycosides, such as myxol 2′-α-l-fucoside, 4-ketomyxol 2′-rhamnoside, and oscillol dichinovoside [14,28]. The glycoside attached to myxol and oscillol is usually one of three methylpentoses: fucose, rhamnose, or chinovose. The carotenogenic pathways are discussed in Section 3.3.1. Most cyanobacteria contain only Chl *a*, whereas some species also contain additional Chl *b*, Chl *d*, Chl *f*, divinyl-chlorophyll *a*, or divinyl-chlorophyll *b* (Table 1).

Algal classification progresses with the developments in phylogenic classification and taxonomic technologies. This review describes both microalgae and macroalgae found in seawater and freshwater without distinction since they share similar pigment compositions. Algae are commonly categorized based on the pigments contained into red algae, brown algae, and green algae, as described below. The carotenoids in algae are highly diverse, and their compositions vary according to divisions and/or classes (Table 1).

In red algae (Rhodophyta), carotenoid composition particularly varies. These algae contain only Chl *a* and can be classified into three types based on carotenoid composition and phylogenetics: an unicellular group, which contains only β-carotene and zeaxanthin (ZEA-type); a macrophytic group, which also includes additional antheraxanthin (ANT-type); and Bangiophyceae and as others, which contain α-carotene and lutein in addition to ZEA-type carotenoids (LUT-type) (Table 1) [15,29]. The carotenogenic pathways are discussed in Section 3.3.2.

Glaucophyta contains Chl *a*, β-carotene, and zeaxanthin as the ZEA-type of red algae (Table 1) [15].

Cryptophyta contains Chl *a*, Chl *c*_2_, α-carotene, zeaxanthin, and alloxanthin (Table 1). The major carotenoid is acetylenic (C≡C) alloxanthin. In addition, it contains α-carotene and its unique acetylenic derivatives, crocoxanthin, and monadoxanthin, which are found only in this division [15]. The carotenogenic pathways are discussed in Section 3.3.2.

**Figure 2:**
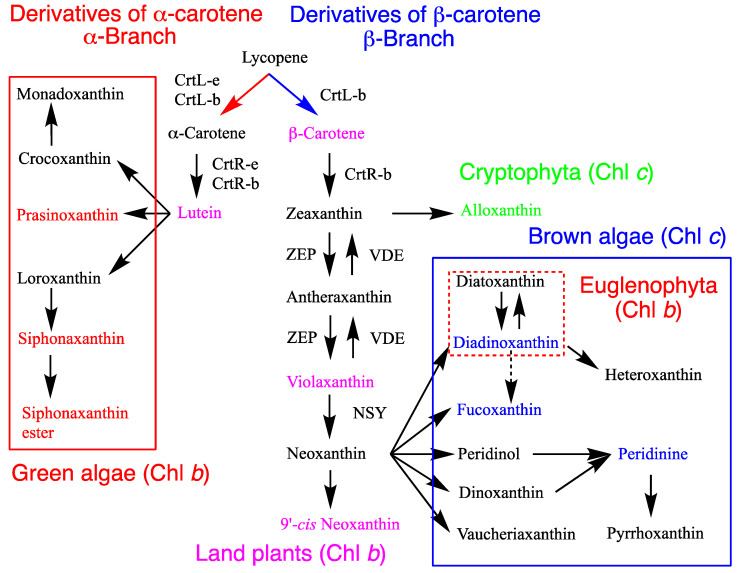
Carotenogenesis pathways and chlorophylls in the chloroplasts of algae and land plants, highlighting key differences and functionally confirmed enzymes involved. The pathways, enzymes, and chlorophyll content vary across algal divisions and classes. This figure is an updated version presented in the previous review [3]. The recently proposed fucoxanthin pathway is referred later.

Brown algae, including Heterokontophyta, Haptophyta, and Dinophyta in this review, contain β-carotene and its derivatives (β-branch carotenoids) as well as Chl *a* and Chl *c* (Table 1, Figure 2). Chl *c* pigments, Chl *c*_1_, Chl *c*_2_, and Chl *c*_3_ are known (Figure 1), and their distribution is specific to divisions and classes (Table 1). Brown algae contain unique acetylenic carotenoids, such as diadinoxanthin and diatoxanthin. Fucoxanthin and its derivatives are found only in four classes of Heterokontophyta (Chrysophyceae, Raphidophyceae, Bacillariophyceae, and Phaeophyceae), Haptophyta, and Dinophyta. Peridinin and its derivatives, as well as P457 [30], are unique to Dinophyta. Both fucoxanthin and peridinin are characterized by their unique structures (Figure 2) and class-specific distributions (Table 1) [2]. The carotenogenic pathways are discussed in Section 3.3.3. Notably, brown algae lack lutein and its derivatives (α-branch carotenoids), whereas Eustigmatophyceae lacks Chl *c*, fucoxanthin, diadinoxanthin, and diatoxanthin but contains β-carotene, violaxanthin, and vaucheriaxanthin.

Green algae, including Euglenophyta, Chlorarachniophyta, and Chlorophyta in this review, contain the same carotenoids, including β-carotene, violaxanthin, 9′-*cis* neoxanthin [12], and lutein, as well as Chl *a* and Chl *b*, all of which are present in land plants (Table 1, Figure 2). Some classes contain additional carotenoids, such as loroxanthin, siphonaxanthin, and prasinoxanthin, which are derivatives of lutein (α-branch carotenoids) and specific to particular classes. The carotenogenic pathways are discussed in Section 3.3.4. Notably, Euglenophyta lacks lutein and its derivatives (α-branch carotenoids) but contains diadinoxanthin and diatoxanthin.

Land plants, such as moss, fern, gymnosperm, and angiosperm, primarily contain only four types of carotenoids: β-carotene, violaxanthin, 9′-*cis* neoxanthin [12], and lutein. These carotenoids are located in the main synthetic pathways and are used for photosynthesis in the pigment-protein complexes (Table 1, Figure 2). The carotenogenic pathways are discussed in Section 3.3.4. Only Chl *a* and Chl *b* are present.

It is important to note that the identifications of some carotenoids may be incomplete because of insufficient analysis. Moreover, some algae names have been updated in response to developments in taxonomic technology and phylogenetic classification [31].

## 3. Carotenogenesis Pathways, Enzymes, and Genes

Carotenogenesis pathways and their enzymes have been primarily examined in cyanobacteria [14] and land plants among the oxygenic phototrophs [2,17,18]. The carotenogenesis pathways and enzyme characteristics for land plants are presented in Figure 2. Algae share common pathways with land plants but also contain additional algae-specific pathways, which have been proposed based on the chemical structures of carotenoids (Figure 2). Some common carotenogenesis genes in algae have been identified through gene homology, whereas many genes and enzymes specific to algal pathways remain unknown (Figure 2). In cyanobacteria, carotenoid compositions are different from those in algae and land plants. Therefore, their carotenogenesis pathways and enzymes are also different from those presented in Figure 2.

### 3.1. Lycopene Synthesis

#### 3.1.1. Isopentenyl Pyrophosphate to Phytoene Synthesis

Isopentenyl pyrophosphate (IPP), a C_5_-compound, is the precursor for isoprenoids, terpenes, quinones, sterols, phytol of chlorophylls and bacteriochlorophylls, and carotenoids. IPP is synthesized via two distinct pathways: the classical mevalonate (MVA) pathway and the alternative, non-mevalonate, 1-deoxy-d-xylulose-5-phosphate (DOXP) pathway [32,33]. In the MVA pathway, acetyl-coenzyme A is converted to IPP through mevalonate, and the genes and enzymes involved have been characterized. This pathway occurs in the cytoplasm of algae and land plants, yeast, animals, and some bacteria, including green filamentous bacteria [32,34]. In contrast, the DOXP pathway was discovered in the 1990s, which involves the conversion of pyruvate and glyceraldehyde into IPP. The DOXP pathway is found in cyanobacteria, the plastids of algae and land plants, and certain bacteria, including purple bacteria, green sulfur bacteria, and *E. coli*. Although carotenoids are synthesized in plastids, the genes responsible for their synthesis are encoded in nuclear DNA. Unique to oxygenic phototrophs, Euglenophyta only contains the MVA pathway, whereas Chlorophyceae only has the DOXP pathway [32,33].

All carotenoids in oxygenic phototrophs contain eight IPP units. Farnesyl pyrophosphate (C_15_) is synthesized from three IPP units. Subsequently, one IPP unit is added to farnesyl pyrophosphate by geranylgeranyl pyrophosphate synthase (CrtE, GGPS) to yield geranylgeranyl pyrophosphate (C_20_). With a head-to-head condensation of the two C_20_-compounds, the first carotene, phytoene (C_40_), is synthesized by phytoene synthase (CrtB, Pys, Psy) using ATP [35,36]. This pathway was confirmed by cloning the genes from two species of purple bacteria, *Rhodobacter capsulatus* and *Cereibacter sphaeroides* (previously *Rhodobacter*), and two species of *Pantoea* [35,36,37]. Among the oxygenic phototrophs, the functions of CrtE and CrtB have also been confirmed. CrtE occurs in *Thermosynechococcus elongates* [38], *Porphyra umbilicalis* [39], *Euglena gracilis* [40], and *Arabidopsis thaliana* [41], and CrtB is present in *Gloeobacter violaceus* [42], *Phaeodactylum tricomutum* [43], *Chlamydomonas reinhardtii* [44], *Haematococcus lacustris* (previously, *pluvialis*) [45], and *Euglena gracilis* [40]. The CrtE and CrtB genes exhibit high sequence homology from bacteria to land plants, respectively [2].

#### 3.1.2. Phytoene to Lycopene Synthesis

Four desaturation steps are needed for the conversion of phytoene to lycopene. Oxygenic phototrophs, including cyanobacteria, algae, and land plants, require four enzymes for this process: phytoene desaturase (plant type) (CrtP, Pds), ζ-carotene desaturase (CrtQ, Zds), ζ-carotene isomerase (Z-ISO), and *cis*-carotene isomerase (CrtH, CrtISO). CrtP catalyzes the first two desaturation steps from phytoene to ζ-carotene through phytofluene. CrtQ then catalyzes two additional desaturation steps from ζ-carotene to lycopene through neurosporene. During desaturation by CrtP, 9,15,9′-*tri-cis* ζ-carotene is produced and subsequently converted to 9,9′-*di-cis* ζ-carotene by Z-ISO. This reaction is also catalyzed with light, nonenzymatically. In addition, during the desaturation by CrtQ, neurosporene and lycopene are isomerized to poly-*cis* forms, followed by isomerization to all-*trans* forms by CrtH. Light is also effective for their photoisomerization to all-*trans* forms, nonenzymatically. The functions of these enzymes have been mainly established in cyanobacteria, green algae, and land plants: CrtP from *Synechocystis* sp. PCC 6803 [46], *Synechococcus elongatus* PCC 7942 [47], *Phaeodactylum tricornutum* [43], *Euglena gracilis* [48], *Chlamydomonas reinhardtii* [49], and *Chromochloris* (previously, *Chlorella*) *zofingiensis* [50]; CrtQ from *Anabaena* sp. PCC 7120 (CrtQa, CrtI-like sequence) [51], *Synechocystis* sp. PCC 6803 (CrtQb, plant Crt*Q*-like) [52], *Phaeodactylum tricornutum* [43] and *Euglena gracilis* [48]; Z-ISO from *Arthrospira* and *Euglena* [53] and *Arabidopsis* and *Zea* [54]; and CrtH from *Synechocystis* sp. PCC 6803 [55,56], *Arabidopsis* [57], and *Lycopersicon* [58]. CrtP of *S. elongatus* PCC 7942 is stimulated by NAD(P) and oxygen as a potential final electron acceptor [59]. CrtQa shows sequence homology with bacterial phytoene desaturase (CrtI) and CrtH, whereas CrtQb shows sequence homology with CrtP. In addition, the genes homologous to Crt*Qa* are not found in cyanobacteria. Therefore, among the oxygenic phototrophs, *Ana-baena* sp. PCC 7120 is the only species with functional CrtQa.

In contrast, the bacterial type requires only one enzyme, phytoene desaturase (bacterial type) (CrtI), to convert from phytoene to lycopene. Notably, this type of CrtI is only found in the primitive cyanobacterium *Gloeobacter violaceus*, whereas the homologous genes of CrtP, CrtQ, and CrtH are not present in the genome [42,60]. Among anoxygenic phototrophs, purple bacteria, green filamentous bacteria, and heliobacteria use CrtI, whereas green sulfur bacteria use CrtP, CrtQ, and CrtH but do not use Z-ISO [10,53]. CrtI shares amino acid sequence homology with CrtP, CrtQ, CrtH, CrtO, and CrtD.

### 3.2. β-Carotene and α-Carotene Synthesis by Lycopene Cyclases

All carotenoids in oxygenic phototrophs are derived from lycopene: dicyclic carotenoids, β-carotene, and α-carotene (β- and α-branch carotenoids, respectively), and their derivatives (Figure 2). In cyanobacteria, exceptionally, myxol methylpentosides and oscillol di-methylpentosides are present as monocyclic and acyclic carotenoids, respectively. C_30_- and C_50_-carotenoids and derivatives of neurosporene are not found in oxygenic phototrophs.

Lycopene is cyclized into either β-carotene through γ-carotene or α-carotene through δ-carotene. Three distinct families of lycopene cyclases have been identified in carotenogenic organisms [14,61,62,63]. One large family includes CrtY, which is present in some bacteria except cyanobacteria and green sulfur bacteria, and CrtL (CrtL-b, Lcy-b), which is found in certain cyanobacteria, algae, and land plants. Lycopene ε-cyclases (CrtL-e, Lcy-e) from green algae and land plants and lycopene β-monocyclases (CrtYm, CrtLm) from bacteria are also included. The amino acid sequences for these lycopene cyclases contain five significantly conserved regions [27,61,64] and have a NAD(P)/FAD-binding motif [65]. Note that the Sandmann group [27,61] and Takaichi group [63] consider these enzymes to belong to the same family, whereas the Bryant group [62] classifies them into two separate CrtY and CrtL families.

Two cyanobacteria also express CrtL-type enzymes. *Synechococcus elongatus* PCC 7942 contains a functional CrtL [66]. *Prochlorococcus marinus* MED4 contains two lycopene cyclases, both of which have sequence homology to CrtL. CrtL-b only exhibits lycopene β-cyclase activity, whereas CrtL-e is a bifunctional enzyme with both lycopene ε-cyclase and lycopene β-cyclase activities. The combination of these two cyclases enables the production of β-carotene, α-carotene and ε-carotene. Both enzymes may have originated from the duplication of a single gene [67]. The characteristics of this CrtL-e differ somewhat from those in land plants [68]. *Prochlorococcus marinus* produces the usual (6*R*)-α-carotene, whereas *Acaryo-chloris marina* MBIC 11017 produces (6*S*)-α-carotene, which displays the opposite chirality to the usual (6*R*)-α-carotene and contains only one CrtL-like gene based on its genome sequence [4]. Among algae, certain CrtL enzymes also have the functions; *Cyanidioschyzon merolae* [69], *Phaeodactylum tricomutum* [43], *Dunaliella salina* [64], and *Haematococcus lacustris* [70]. CrtL-b and CrtL-e from both *Porphyra umbilicalis* [71] and *Chloromochloris zofingliensis* [72] also have the functions.

The second family of lycopene cyclases includes a heterodimer consisting of CrtYc and CrtYd found in bacteria, a fused monomer (CrtYc-Yd) present in both bacteria and archaea, and a fused bifunctional CrtYB found in fungi [73,74,75,76], but not in phototrophs.

A new family of functional lycopene cyclases, CruA, has been found in *Chlorobaculum tepidum* (green sulfur bacterium). Homologous genes, CruA and CruP, have also been found in the genome of *Synechococcus* sp. PCC 7002, with their primary product being γ-carotene when expressed in lycopene-producing *E. coli* [62]. However, Bradbury et al. [76] were not able to detect CruP activity in *Synechococcus* sp. PCC 7002 in lycopene-producing *E. coli*. More recently, Sugiyama et al. [77] demonstrated that CruA from *Arthrospira platensis* exhibits lycopene cyclase activity in lycopene-producing *E. coli*. In addition, Xiong et al. [78] demonstrated that CruA from *Synechocystis* sp. PCC 6803 exhibits lycopene cyclase activity in the CruA-less mutant of *Synechococcus* sp. PCC 7002, which requires bound Chl *a*; however, the functions of bound Chl *a* are unclear.

The homologous genes CruA and CruP are widely distributed across the genomes of certain cyanobacteria; however, the confirmation of the activities of these CruA- and CruP-like genes remains limited, as noted above. Phylogenetic analysis of functional CruA, CruP, and CrtL-type lycopene cyclases, along with their homologs in cyanobacteria, revealed two distinct clusters [53]; however, further studies are needed to determine the distribution and functionality of lycopene cyclases such as CrtL-, CruA-, and CruP-like types, in cyanobacteria.

The distribution of α-carotene and its derivatives (α-branch carotenoids) is restricted to certain red algae, green algae, and land plants (Table 1). The characteristics of the genes and enzymes of CrtL-e have been investigated, and they show sequence homology with CrtL*-b*. Lycopene is initially converted to δ-carotene by CrtL-e and then to α-carotene by CrtL-b. γ-Carotene produced by CrtL-b is not a suitable substrate for CrtL-e [63,68]. Their functions have been confirmed in red alga *Porphyra umbilicalis* [71], green alga *Chloromochloris zofingliensis* [72], and some species of land plants [68],

### 3.3. Xanthophyll Synthesis

#### 3.3.1. Cyanobacteria

**Figure 3:**
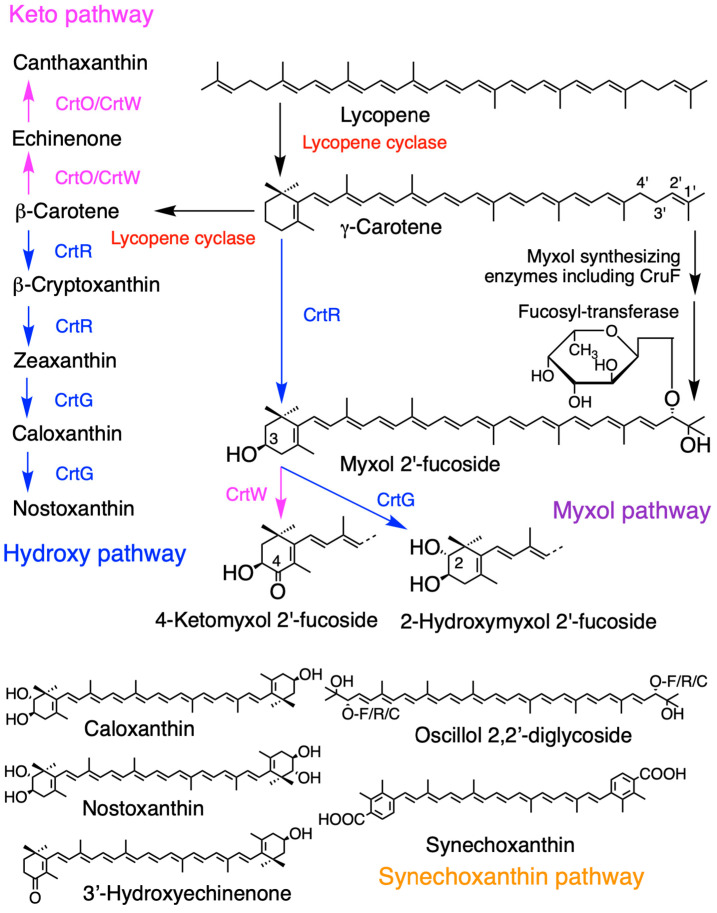
Major carotenogenesis pathways in cyanobacteria along with the functionally confirmed enzymes involved. These pathways and enzymes vary across cyanobacterial species. Two lycopene cyclases, CruA and CrtL, have been identified. The glycoside attached to myxol and oscillol may be fucose, rhamnose, or chinovose. This figure is an updated version presented in the previous review [3].

Most cyanobacteria produce zeaxanthin, whereas some produce both zeaxanthin and nostoxanthin (Figure 3, hydroxy pathway). The hydroxy group is first introduced into the C-3 and C-3′ positions of β-carotene by β-carotene hydroxylase (CrtR) to produce zeaxanthin via β-cryptoxanthin. Subsequently, at the C-2 and C-2′ positions of zeaxanthin, the hydroxy group is introduced by 2,2′-β-hydroxylase (CrtG) to produce nostoxanthin via caloxanthin [16,79,80,81,82,83]. The same enzymes, CrtR and CrtG, can also introduce hydroxy groups to deoxymyxol and myxol to produce myxol and 2-hydroxymyxol, respectively (myxol pathway) [80,83]. Thus, CrtR and CrtG are involved in two different pathways. CrtR is a non-heme di-iron enzyme, whereas CrtG is a cytochrome P450-type enzyme, and they do not share sequence homology [14].

Cyanobacteria contain some ketocarotenoids, including echinenone, canthaxanthin, 3′-hydroxyechinenone, astaxanthin, and 4-ketomyxol. Two distinct β-carotene ketolases, CrtO and CrtW, have been identified. Although both enzymes catalyze the ketolation of the β-end group, they have different characteristics (Figure 3, keto pathway) [14]. CrtO is almost twice the size of CrtW and shows little amino acid sequence homology with CrtW. CrtO usually converts β-carotene to echinenone; however, the final product of canthaxanthin is low or absent in *Synechocystis* sp. PCC 6803, *Anabaena* sp. PCC 7120, and *Gloeobacter violaceus* [42,82,84,85]. In contrast, CrtW introduces one or two keto groups into β-carotene, zeaxanthin, and myxol to produce canthaxanthin, astaxanthin, and 4-ketomyxol, respectively, in *Anabaena* sp. PCC 7120, *Nostoc punctiforme*, and *Gloeobacter violaceus* [42,60,82,84,86]. Taken together, these ketolases function in different pathways, β-carotene and myxol, depending on the species [14,82].

The pathways and the enzymes involved in the production of the right half structure of myxol 2′-methylpentoside remain unclear (Figure 3, myxol synthesizing enzymes in myxol pathway) [2,14]; however, two enzymes from *Synechococcus* sp. PCC 7002 have been functionally confirmed: carotene 1′,2′-hydratase (CruF), and glycosyltransferase (CruG) [87].

Synechoxanthin contains an aryl end group with a carboxy group, and both structures are rare in carotenoids (Figure 3, synechoxanthin pathway). Its chemical structure was identified in 2008 [88]. Some cyanobacteria, such as *Synechococcus* sp. PCC 7002, *Synechocystis* sp. PCC 6803, and *Anabaena* sp. PCC 7120 produces this carotenoid; however, its distribution in cyanobacteria has not been fully examined. It is produced from β-carotene, and two biosynthetic enzymes (CruE and CruH) have been functionally identified, although one is unknown [89].

#### 3.3.2. Red Algae

**Figure 4:**
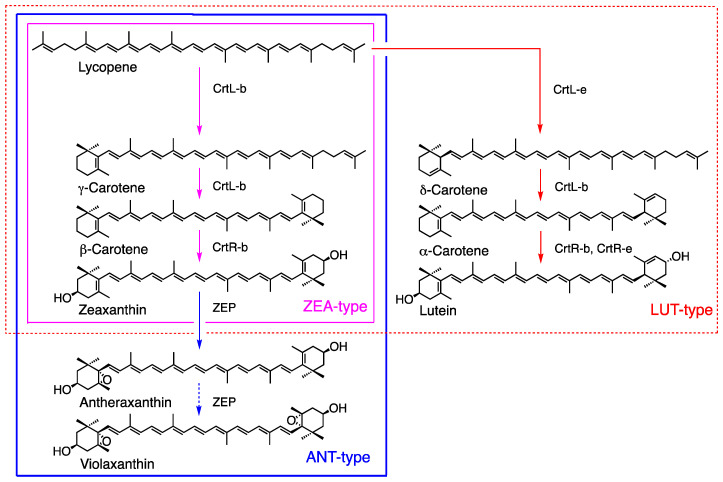
Carotenogenesis pathways in red algae categorized into three types based on carotenoids and phylogeny [15].

Red algae (Rhodophyta) produce a wide variety of carotenoids and may be categorized into three types based on carotenoid composition and phylogenetics, including the ZEA-type (Figure 4, magenta line), ANT-type (blue line), and LUT-type (red line) as described in Section 2 (Table 1) [15,29]. The major carotenoids of the ZEA-type are β-carotene and zeaxanthin, and this type contains homologous genes, such as CrtL-b and CrtR-b, which are involved in zeaxanthin synthesis. The ANT-type includes antheraxanthin in addition to the ZEA-type and features the enzyme of zeaxanthin epoxidase ZEP for antheraxanthin synthesis, whose function is confirmed in *Madagascaria erythrocladioides* [90]. Antheraxanthin is the major carotenoid, whereas violaxanthin is either absent or present in minor amounts, and it may have an important role in photosynthesis. The LUT-type is characterized by the presence of α-carotene and lutein and includes CrtL-e and CrtR-e for lutein biosynthesis (Figure 4). The function of the P450-type β-carotene hydroxylase (CYP97) has been confirmed in *Porphyra umbilicalis* [91].

Cryptophyta contains α-carotene, zeaxanthin, and alloxanthin (Table 1). In organic chemistry, the C-7,8 and C-7′,8′ double bonds of zeaxanthin can be oxidized to form the triple bond (acetylenic group, C≡C) present in alloxanthin [17]. Therefore, in Cryptophyta, alloxanthin may be enzymatically synthesized from zeaxanthin since there are no epoxy and allenic carotenoids as diadinoxanthin (Figure 2).

#### 3.3.3. Brown Algae

Major carotenoids in brown algae, including Heterokontophyta, Haptophyta, and Dinophyta, are β-carotene, zeaxanthin, diadinoxanthin, fucoxanthin, and peridinin, depending on the divisions and classes. Notably, the α-branch carotenoids are absent. Little is known regarding the carotenogenesis pathways of brown algae; however, some have been proposed based on the chemical structures of the carotenoids present, and the functions of some enzymes have been confirmed as below (Table 1, Figure 2).

In the cell-free preparations of *Amphidinium carterae* (Dinophyta), ^14^C-labeled zeaxanthin was converted into allenic neoxanthin, which was then converted into acetylenic (C≡C) diadinoxanthin and C_37_ peridinin (Figure 2). Moreover, the three carbon atoms at C-13′,14′, and 20′ of peridinin are eliminated from neoxanthin (C-13,14,20) [92,93]. Allenic carotenoids (C=C=C) are rare in algae. Based on their chemical structures, all-*trans* neoxanthin may be converted into fucoxanthin, diadinoxanthin, dinoxanthin, peridinin, and vaucheriaxanthin; however, the specific pathways and enzymes involved are still unclear (Figure 2). Diadinoxanthin and diatoxanthin also contain a triple bond (acetylene group) and may be synthesized from the epoxy and allene groups of neoxanthin since they are asymmetric structures. Consequently, the formation of the acetylene group in diadinoxanthin in brown algae and alloxanthin in Cryptophyta occur differently (Figure 2).

**Figure 5:**
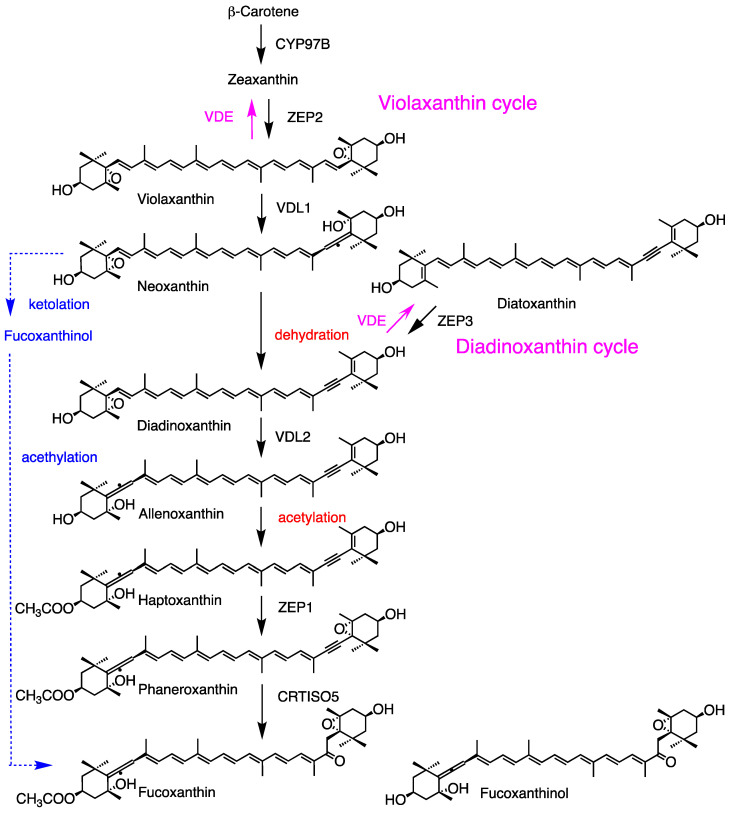
Carotenogenesis pathway from β-carotene to fucoxanthin, as well as violaxanthin and diadinoxanthin cycles in the brown alga *Phaeodactylum tricornutum*. VDE is activated by high-light conditions. The dotted blue line highlights a simple pathway (Figure 1). The illustrated structures of fucoxanthin and fucoxanthinol are reversed for the right and left sides according to the IUPAC-IUB semi-systematic rule.

For fucoxanthin synthesis, a simplified biosynthetic pathway involving the ketolation and acetylation of *trans* neoxanthin has been proposed based on chemical structures (Figure 5, blue line); however, the specific genes and enzymes have not yet been identified. Recently, an alternative pathway has been discovered in *Phaeodactylum tricomutum* (Figure 5). In this pathway, β-carotene is hydroxylated to zeaxanthin by the P450-type enzyme PtCYP97B2 [94]. Zeaxanthin and diatoxanthin are epoxidated to violaxanthin and diadinoxanthin by the epoxidases PtZEP2 and PtZEP3, respectively [95,96]. Subsequently, both violaxanthin and diadinoxanthin are converted back to zeaxanthin and diatoxanthin, respectively, by the same violaxanthin de-epoxidase (PtVDE), which is activated under high-light conditions [97]. These processes are known as the violaxanthin cycle and diadinoxanthin cycle, respectively [98]. The violaxanthin de-epoxidase-like1 (PtVDL1) enzyme from *P. tricumutum* and some related species exhibits neoxanthin synthase activity in violaxanthin-producing *E. coli*; however, its homologous gene is absent in land plants [97,99]. Neoxanthin is then dehydrated to diadinoxanthin by an unknown dehydratase. Diadinoxanthin is converted to allenoxanthin by violaxanthin de-epoxidase-like2 (VDL2), whose function is confirmed using a green mutant of *P. tricumutum* [100]. Allenoxanthin is subsequently acetylated to form haptoxanthin. The final hydration or ketolation step is catalyzed by PtCrtISO5 [101]. The specific dehydrase and acetylase involved have not yet been unidentified. In addition, homologous genes to *VDL1* and *VDL2* are found only in a limited number of brown algal species. Therefore, the universality of this pathway for fucoxanthin production in brown algae has not yet been confirmed.

#### 3.3.4. Green Algae

Green algae encompass several divisions, such as Euglenophyta, Chlorarachniophyta, and Chlorophyta (Table 1). Their carotenoids primarily include β-carotene, zeaxanthin, violaxanthin, 9′-*cis* neoxanthin, lutein, and lutein derivatives. Functionally confirmed enzymes have mainly been identified in Chlorophyceae, including *Chlorella*, *Chromochloris*, *Chlamydomonas*, *Dunaliella*, and *Haematococcus* for CrtB, CrtP, CrtL-b, CrtR-b, ZEP, VED, and CrtW for the β-branch carotenoids. The α-branch carotenoids, such as siphonaxanthin [13], loroxanthin, prasinoxanthin, and monadoxanthin, may be derived from lutein based on their chemical structures, although the specific pathways and enzymes remain unknown (Figure 2, Table 1). Recently, an intermediate of siphonaxanthin has been identified, and siphonaxanthin may be synthesized from lutein via loroxanthin and/or deoxysiphonaxanthin [102]. Functionally confirmed enzymes include CrtR-b from *Haematococcus lacustris* (previously, *pluvialis*) [103], ZEP from *Chlamydomonas reinhardtii* [104], and VDE from *Mantonilla squamata* [105].

Under stressful conditions, including high-light irradiation, UV irradiation, and/or nutrient stress, some Chlorophyceae, such as *Haematococcus*, *Chlorella*, and *Scenedesmus*, accumulate ketocarotenoids, such as canthaxanthin and astaxanthin [106]. These carotenoids are synthesized by a combination of CrtR-b and β-carotene ketolase (CrtW, BKT) [23,107,108,109,110,111]. Usually, the substrate of CrtR-b hydroxylase is a β-end group (β-carotene), but the keto-β-end group (canthaxanthin) is unsuitable. The substrate of CrtO ketolase is the β-end group, but the hydroxy β-end group (zeaxanthin) is unsuitable, whereas those of CrtW are both β-end and hydroxy-β-end group [82]. The pathways from β-carotene to astaxanthin may depend on the characteristics of enzymes, species, and culture conditions [112]. Notably, while the β-carotene ketolase in *Haematococcus lacustris* (previously, *pluvialis*) and *Chromochloris* (previously, *Chlorella*) *zofingiensiswas* was initially named CrtO [107,109,110], it is actually a CrtW-type, not a CrtO-type, based on amino acid sequences.

Land plants evolved from green algae, including mosses, ferns, gymnosperms, and angiosperms. They primarily contain only four types of carotenoids: β-carotene, violaxanthin, 9′-*cis* neoxanthin [12], and lutein. These carotenoids are located in the main synthetic pathways (Figure 2).

Hydroxy groups are introduced into β-carotene to produce zeaxanthin by β-carotene hydroxylase (CrtR, CrtR-b, BCH). β-Carotene is hydroxylated mainly by the nonheme di-iron enzymes, BCH1 and BCH2 (CrtR-b), to produce zeaxanthin, whereas α-carotene is primarily hydroxylated by the cytochrome P450-type enzymes, CYP97A3 for the β-end group and CYP97C1 for the β- and ε-end groups, which results in the production of lutein [113]. Epoxy groups are introduced into zeaxanthin by zeaxanthin epoxidase (ZEP) to produce violaxanthin via antheraxanthin. Under high-light conditions, violaxanthin is converted back to zeaxanthin by violaxanthin de-epoxidase (VDE). This process is known as the violaxanthin cycle, as described above. One end group of violaxanthin is converted to an allene group in neoxanthin by neoxanthin synthase (NSY). Three proteins from land plants and one from *P. tricumutum* [97,99] have been reported to have NSY activity; however, their homologous genes are not distributed in all land plants and brown algae. Since neoxanthin in chloroplasts exists exclusively in the 9′-*cis* form, unknown 9′-isomerase for all *trans* neoxanthin to 9′-*cis* neoxanthin should be present [12].

**Figure 6:**
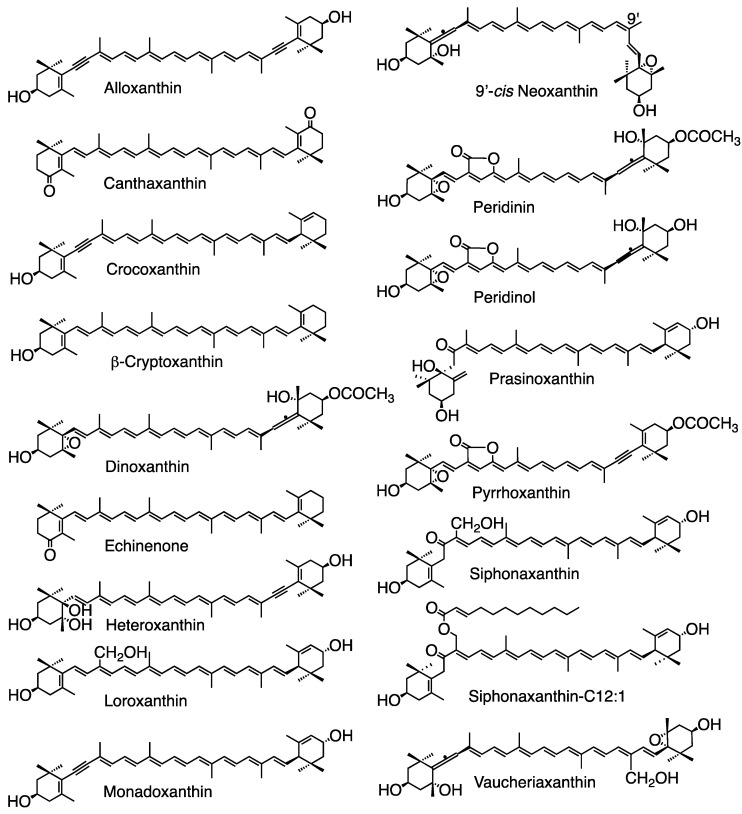
Structures of some carotenoids mentioned in this review.

The structures of the carotenoids are also shown in Figure 6.

## 4. Function of Carotenoids

For photosynthesis, most carotenoids and chlorophylls are bound to peptides to form the photosynthetic pigment-protein complexes and super complexes located in the thylakoid membrane of phototrophs. Certain complexes have been isolated from cyanobacteria, some algae, and land plants, such as photosystem I (PSI), light-harvesting complex I (LHCI), PSI-LHCI super complex, photosystem II (PSII), light-harvesting complex II (LHCII), PSII-LHCII super complex, and the cytochrome *b*_6_*f* complex between PSII and PSI. Their 3D structures, characteristics, and functions, including excitation-energy transfer and pigment compositions, have been examined. The primary functions of carotenoids are light-harvesting and then transferring the energy to chlorophylls, the protection of the chlorophylls and peptides from high-light radiation [114], and the assembly and stability of the pigment-protein complexes as described below. Fucoxanthin-chlorophyll *a/c*-proteins (FCP) from brown algae [115], violaxanthin-chlorophyll-*a*-proteins (VCP) from *Nannochloropsis* (Eustigmatophyceae) [116], PSI-LHCI supercomplex from *Chlorella sorokiniana* [117], and PSII-LHCII supercomplex from *Chaetoeros gracilis* [118] have been isolated and characterized.

β-Carotene is presented in most divisions of the PSI and LHCI as well as the PSII and core LHCII. Zeaxanthin is especially present in some red algae of the LHCI. Meanwhile, in the peripheral LHCII, the bound carotenoids are heterogeneous depending on the classes. The major carotenoids (Figure 2, Table 1) are alloxanthin (Cryptophyta), fucoxanthin (Chrysophyceae, Raphidophyceae, Bacillariophyceae, and Haptophyta), diadinoxanthin and vaucheriaxanthin (Xanthophyceae), violaxanthin and vaucheriaxanthin (Eustigmatophyceae), peridinin (Dinophyta), diadinoxanthin (Euglenophyta), siphonaxanthin (Chlorophyceae and Ulvophyceae), and lutein, violaxanthin, and 9′-*cis* neo-xanthin (land plants). β-Carotene in both PSI and PSII may exert protective functions, and carotenoids in the peripheral LHCII may have light-harvesting functions. Myxol glycosides and some carotenoids are located in the cytoplasmic membrane, stroma, or lumen to protect from the high-light radiation in phototrophs, whereas astaxanthin is accumulated in the cytoplasm of certain green algae under high-light conditions.

The keto groups at C-8 of fucoxanthin [119], siphonaxanthin [120,121], and prasinoxanthin [122], which are found only in algae, represent the single-bond *trans*-conformation for the conjugated double bond (Figure 2). From the femtosecond time-resolved fluorescence spectroscopy of the purified carotenoids in organic solvents and the LHC in solution, these ketocarotenoids, along with peridinin, transfer the harvested energy highly efficiently from the S_1_ state, but not the S_2_ state, of carotenoids to chlorophylls. A comparison of the structures of other carotenoids revealed that these keto groups are essential for high transfer efficiency [123,124]. These ketocarotenoids may primarily have light-harvesting functions.

The water-soluble peripheral LHC of peridinin-chlorophyll-protein (PCP) isolated from *Amphidinium carterae* and *Symbiodinium* sp. CS-156 (Dinophyta) exhibits a trimeric structure, and the monomer contains eight peridinin and two Chl *a* molecules [125,126]. The water-soluble orange carotenoid protein (OCP) isolated from the cyanobacterium *Arthrospira maxima* forms a homodimer with two 3′-hydroxyechinenone molecules [127]. OCP is also produced in some cyanobacteria, and its function might regulate the energy dissipation from phycobilisomes to PSII [128,129].

The xanthophyll cycle has a role in protecting from the potentially harmful effects of excess light by enhancing the dissipation of excess energy as heat. It may be divided into the violaxanthin and diadinoxanthin cycles. The violaxanthin cycle is the cyclical interconversion of violaxanthin, antheraxanthin, and zeaxanthin in green algae and land plants, whereas the diadinoxanthin cycle is that of diadinoxanthin and diatoxanthin in brown algae, Heptophyta, Dinophyta, and Euglenophyta (Figure 2, Table 1) [98,130]. These have been primarily investigated in land plants. Zeaxanthin epoxidase (ZEP) converts zeaxanthin to violaxanthin through antheraxanthin during biosynthesis. Violaxanthin is found in the peripheral LHC of PSII. Under high-light conditions, the lumen inside of the thylakoid is acidified by proton movement to the lumen, and then violaxanthin de-epoxi-dase (VDE) in the lumen is activated by acidic pH and ascorbate. VDE catalyzes the de-epoxidation of violaxanthin to zeaxanthin through antheraxanthin. Zeaxanthin dissipates excess energy from excited chlorophylls. ZEP from Chlorophyceae *Chlamydomonas reinhardtii* [104,131] and *Chromochloris* (previously, *Chlorella*) *zofingiensis* [132], and VDE from Pracinophyceae *Mantonilla squamata* [105] and *C. reinhardtii* [131] have been functionally confirmed. Similarly, the diadinoxanthin cycle occurs in Heterokontophyta, Haptophyta, and Dinophyta, which contain diadinoxanthin and diatoxanthin. The diadinoxanthin and diatoxanthin contents are normally not low, and a portion may be used for the diadinoxanthin cycle. For the brown alga of *Phaeodactylum tricomutum*, functional zeaxanthin and diatoxanthin cycles are discussed above (Figure 5, Section 3.3.3).

Certain carotenoids are essential for the assembly and structural stability of pigment-protein complexes, such as *Synechocystis* sp. PCC 6803 and *Thermosynechococcus elongatus* (cyanobacteria) [133,134], whereas lutein is essential for the LHCII of land plants.

Water-soluble astaxanthin-binding glycoprotein (AstaP) from *Coelastrella astaxanthina* has been characterized. Its homologs are widely distributed in Scenedesmaceae. They are accumulated under stress conditions, such as high-salt concentration and high-light irradiation. They may have scavenging activity for reactive oxygen species, particularly ^1^O_2_, and may function as a sunscreen [135,136].

Furthermore, under high-light, UV-A, or UV-B radiation, ketocarotenoids, such as astaxanthin and canthaxanthin, are accumulated in green algae and cyanobacteria for protection. In the case of the cyanobacterium *Chlorogloeopsis fritschii* under low level UV-B radiation, the expression levels of carotenogenesis genes of CrtW, CrtR, *Cru*F, and CruG (Figure 3) are increased [137]. Zeaxanthin is accumulated in the cytoplasmic membranes of *Synechococcus elongatus* grown under high-light conditions for protection from light [138]. Astaxanthin esters are also accumulated in oil drops in the cytoplasm of *Haematococcus* under high-light conditions.

## 5. Conclusions

Oxygenic phototrophs, including cyanobacteria, algae, and land plants, necessarily contain chlorophylls and carotenoids for photosynthesis. Their distribution is division and class-specific. Cyanobacteria contain dicyclic carotenoids (β-carotene, zeaxanthin, and echinenone) and monocyclic ones (myxol glycoside). Red algae contain diverse carotenoids. Brown algae contain only β-branch carotenoids (β-carotene, diadinoxanthin, fucoxanthin, and peridinin). Green algae contain β-carotene, violaxanthin, and α-branch carotenoids (lutein, siphonaxanthin, and prasinoxanthin). The carotenogenesis genes and enzymes have been characterized. Main biosynthetic pathways are similar, and specific carotenoids are synthesized from branched pathways. Most carotenoids bound to protein to produce pigment-protein complexes, such as PSI, PSII, LHCI, and LHCII, for light-harvesting and photoprotection functions and the assembly of pigment-protein complexes.

## Figures and Tables

**Table 1:** Distribution of carotenoids and chlorophylls in oxygenic phototrophs. This table is an updated version presented in the previous review [3] ^a,b^.

Division	Carotene ^ c^		Xanthophyll ^ c^											Chlorophyll ^ d^		
**Class**	**β**	**α**	**Ze**	**Vi**	**Ne**	**Da**	**Dd**	**Fx**	**Va**	**Lu**	**Lo**	**Sx**	**Others**	***a***	***b***	***c***
Cyanophyta	H	L	H										No (L), Ec (H), OH-Ec (L), My (H), Sy (L), Ax (L)	H	L	
Glaucophyta	H		H											H		
**Rhodophyta (red algae)**																
ZEA-type	H		H											H		
ANT-type	H		H										An (H)	H		
LUT-type	H	L	L							H				H		
Cryptophyta	L	H	L										Al (H), Cr (L), Mo (L)	H		*c*_2_
**Heterokontophyta (brown algae)**																
Chrysophyceae	H		L	L		L	L	H						H		*c*_1_, *c*_2_
Raphidophyceae	H		H	L		L	L	H						H		*c*_1_, *c*_2_
Bacillariophyceae	H		L			L	L	H						H		*c*_1_, *c*_2_, *c*_3_
Phaeophyceae	H		H	H		L	L	H					An (L)	H		*c*_1_, *c*_2_, *c*_3_
Xanthophyceae	H		L		L	H	H		L				He (L), Va-FA (L)	H		*c*_1_, *c*_2_
Eustigmatophyceae	H		L	H					L				Va-FA (L), An (L)	H		
Haptophyta	H		L			L	H	H	L				Fx-FA (L)	H		*c*_1_, *c*_2_
Dinophyta	L		L	L		L	H	L					Pe (H), P457 (L)	H		*c*_2_
Euglenophyta	H		L		L	L	H						Ax (L)	H	H	
Chlorarachniophyta	H	L	L	L	L					L	L		Lo-FA (L)	H	H	
**Chlorophyta (green algae)**																
Prasinophyceae	H	L	L	H	H					L	L	H	Pr (L), Lo-FA (L), Sx-FA (H), An (L), Ax (L)	H	H	
Chlorophyceae	H	H	L	H	H					H	L	L	Sx-FA (L), An (L), Ax (L)	H	H	
Ulvophyceae	H	L	L	H	H					L	L	L	Sx-FA (H), An (L), Ax (L)	H	H	
Trebouxiophyceae	H	L	L	H	H					H			An (L)	H	H	
Charophyceae	H	L	L	H	H					H			An (L)	H	H	
Land Plants	H	L	L	H	H					H			An (L)	H	H	

^a^ Contents: H, Major catenoid or chlorophyll in most species of the division or class; L, Low content in most species or major carotenoid in limited species; Blank, absent. ^b^ Type of carotenoids: Black, β-Branch carotenoids; Red, α-Branch carotenoids. ^c^ Abbreviation of carotenoids: α, α-carotene; β, β-carotene; Al, alloxanthin; An, antheraxanthin; Ax, astaxanthin; Cr, crocoxanthin; Da, diatoxanthin; Dd, diadinoxanthin; Ec, echinenone; -FA, fatty acid ester; Fx, fucoxanthin; He, heteroxanthin; Lo, loroxanthin; Lu, lutein; Mo, monadoxanthin; My, myxol 2′-glycosides and 2,2′-oscillol diglycosides; Ne, neoxanthin; No, nostoxanthin; OH-Ec, 3′-hydroxyechinenon; Pe, peridinin; Pr, prasinoxanthin; Sx, siphonaxanthin; Sy, synechoxanthin; Va, vaucheriaxanthin; Vi, violaxanthin; Ze, zeaxanthin. ^d^ Chlorophylls: Some cyanobacteria contain Chl *d*, Chl *f*, divinyl-chlorophyll *a*, or divinyl-chlorophyll *b* [4,5]. Chl c is divided into Chl *c*_1_, Chl *c*_2_, and Chl *c*_3_ (Figure 1), and their types are also indicated. Their contents are “H”.

## Data Availability

Not applicable.

## Abbreviations

Abbreviation of carotenogenesis enzymes (parentheses indicate other names).

CrtB (Pys, Psy)	phytoene synthase
CrtE (GGPS)	geranylgeranyl pyrophosphate synthase
CrtG	2,2′-β-hydroxylase
CrtH (CrtISO)	*cis*-carotene isomerase
CrtI	phytoene desaturase (bacterial type)
CrtL (CrtL-b, Lcy-b, CrtY)	lycopene β-cyclase
CrtL-e (Lcy-e)	lycopene ε-cyclase
CrtO	β-carotene ketolase
CrtP (Pds)	phytoene desaturase (plant type)
CrtQ (Zds)	ζ-carotene desaturase
CrtR (CrtR-b, BCH)	β-carotene hydroxylase
CrtR-e	ε-carotene hydroxylase
CrtW (BKT)	β-carotene ketolase
CruA	lycopene cyclase
CruF	carotene 1′,2′-hydratase
CruG	glycosyltransferase
CruP	lycopene cyclase
NSY	neoxanthin synthase
VDE	violaxanthin de-epoxidase
VDL1	violaxanthin de-epoxidase-like1 neoxanthin synthase
VDL2	violaxanthin de-epoxidase-like2 allenoxanthin synthase
ZEP (ZEP2)	violaxanthin epoxidase
ZEP3	diadinoxanthin epoxidase
Z-ISO	ζ-carotene isomerase
